# Comparison of the inoculum effect of in vitro antibacterial activity of Imipenem/relebactam and Ceftazidime/avibactam against ESBL-, KPC- and AmpC-producing *Escherichia coli* and *Klebsiella pneumoniae*

**DOI:** 10.1186/s12941-023-00660-5

**Published:** 2023-12-10

**Authors:** Xueting Wang, Luying Xiong, Yuan Wang, Kai Yang, Tingting Xiao, Xiaohui Chi, Tao Chen, Yanzi Zhou, Ping Lu, Dilimulati Dilinuer, Pin Shen, Yunbo Chen, Yonghong Xiao

**Affiliations:** 1 Collaborative Innovation Center for Diagnosis and Treatment of Infectious Diseases, State Key Laboratory for Diagnosis and Treatment of Infectious Diseases, The First Affiliated Hospital, College of Medicine, Zhejiang University, Hangzhou, China; 2 Fuwai Yunnan Cardiovascular Hospital, Kunming, China; 3 Department of Rheumatology, Affiliated Hangzhou First People’s Hospital, School of Medicine, Westlake University, Hangzhou, China; 4 Jinan Microecological Biomedicine Shandong Laboratory, Jinan, China; 5 Research Units of Infectious Disease and Microecology, Chinese Academy of Medical Sciences, Beijing, China

**Keywords:** Imipenem/relebactam, Ceftazidime/avibactam, Inoculum effect, ESBL, KPC, AmpC, In vitro PK/PD study

## Abstract

**Objective:**

To evaluate effect of inoculum size of extended-spectrum β-Lactamase (ESBL)-producing-, AmpC-producing-, and KPC-producing *Escherichia coli* and *Klebsiella pneumoniae* on the in vitro antibacterial effects of imipenem/relebactam (IMR) and ceftazidime/avibactam (CZA).

**Methods:**

We compared the impact of inoculum size on IMR and CZA of sixteen clinical isolates and three standard isolates through antimicrobial susceptibility tests, time-kill assays and in vitro PK/PD studies.

**Results:**

When inoculum size increased from 10^5^ to 10^7^ CFU/mL, an inoculum effect was observed for 26.3% (5/19) and 52.6% (10/19) of IMR and CZA, respectively; time-kill assays revealed that the concentration of CZA increased from ≥ 4 × MIC to 16 × MIC to reach 99.9% killing rate against *K. pneumoniae* ATCC-BAA 1705 (KPC-2-, OXA-9- and SHV-182-producing) and 60,700 (SHV-27- and DHA-1-producing). While for IMR, a concentration from 1 × MIC to 4 × MIC killed 99.9% of the four strains. When the inoculum size increased to 10^9^ CFU/mL, neither IMR nor CZA showed a detectable antibacterial effect, even at a high concentration. An in vitro PK/PD study revealed a clear bactericidal effect when IMR administered as 1.25 g q6h when inoculum size increased.

**Conclusion:**

An inoculum effect on CZA was observed more frequent than that on IMR. Among the β-lactamase-producing strains, the inoculum effect was most common for SHV-producing and KPC-producing strains.

**Supplementary Information:**

The online version contains supplementary material available at 10.1186/s12941-023-00660-5.

## Background

Production of β-lactamases is the main reason underlying the antimicrobial resistance (AMR) of Gram-negative bacteria; in addition, the selection of β-lactams is closely related to the frequency and evolution of AMR [1]. *Enterobacteriaceae* producing extended-spectrum-β-lactamases (ESBL) and carbapenemases are the main source of multi-drug resistant, and even pan-drug resistant, bacteria worldwide. While the number of treatments is limited, making effective treatment a clinical challenge.

Imipenem/relebactam (IMR) and ceftazidime/avibactam (CZA) are new β-lactam/β-lactamase inhibitor combinations (BLBLIs) approved by the FDA for the treatment of multi-drug resistant bacterial infections. CZA shows potent in vitro activity against ESBL-, KPC- and AmpC-producing *Enterobacteriaceae*, with susceptibility rates of 100%, 96.6% and 95.1%, respectively; IMR also shows good in vitro activity against ESBL-, AmpC-, and KPC-producing strains, with susceptibility rates of 100%, 95.1% and 84.5%, respectively [2].

However, the clinical efficacy may be poor even though the strains are sensitive in an in vitro antimicrobial susceptibility test. Differences in the bacterial load have an impact on the choice of drugs and the dosing regimens; in particular, some antibiotics are susceptible to an inoculum effect (defined as attenuated antibacterial activity with increased inoculum size), which has an impact on treatment of infections. The recommended standard bacterial inoculum for the microbroth method is 1–5 × 10^5^ CFU/mL [3]; however, the bacterial load varies greatly according to the type and location of an infection. With respect to urinary tract infections (UTIs), the bacterial load is usually low, at about 10^5^–10^6^ CFU/mL, while in intra-abdominal infections (IAIs) the bacterial load can be as high as 10^8^–10^9^ CFU/mL, and that in meningitis can reach 10^9^ CFU/mL [4, 5]. A series of studies have examined the inoculum effect, and found that carbapenems are less affected than classical BLBLIs (such as piperacillin/tazobactam) and cephalosporins [6, 7]. In clinical practice, carbapenems are recommended for the treatment of severe infections. Studies show that there is a difference between the clinical cure rates of CZA when used to treat complicated urinary tract infections (cUTIs) and complicated intra-abdominal infections (cIAIs) (92% *vs.* 80%, respectively) [8]; the cure rates of IMR are similar (97.1% *vs*. 96.3%, respectively) [9, 10]. These findings may be related to the inoculum effect. However, there is no consensus about the impact of the inoculum effect on treatment efficacy.

The purpose of this study was to use antimicrobial susceptibility tests, time-kill assays, and in vitro PK/PD studies to evaluate the in vitro inoculum effect of ESBL-, KPC-, and AmpC- producing *E. coli* and *K. pneumoniae* on IMR and CZA, thereby providing a reference for clinical application.

## Material and methods

### Strains

In this study, four clinical isolates of *E. coli* and four clinical isolates of *K. pneumoniae* were examined, as well as standard strains *E. coli* ATCC 25922, *K. pneumoniae* ATCC-BAA 1705 (KPC-2-, OXA-9- and SHV-182-producing), and 700,603 (SHV-18-, OXA-2- and OKP-B-7-producing). Bacterial genomic DNA was extracted using the QIAamp DNA mini kit and subjected to whole genome sequencing to identify β-lactamase types (Table 1).

**Table 1:** β-lactamase genotypes, and the effects of different inoculum sizes on the antibacterial MICs of CZA and IMR against *E. coli* and *K. pneumoniae*

Isolate/species		β-lactamase genes	MIC (mg/L) in the presence of different inocula (CFU/mL)											
			CZA						IMR					
			AVI = 4 mg/L			AVI = 8 mg/L			REL = 4 mg/L			REL = 8 mg/L		
			10^5^	10^7^	10^9^	10^5^	10^7^	10^9^	10^5^	10^7^	10^9^	10^5^	10^7^	10^9^
*E. coli*	ATCC 25922	–	0.06/4	0.25/4	> 512/4	0.03/8	0.125/8	64/8	0.125/4	0.25/4	> 512/4	0.007/8	0.03/8	64/8
	56,706*	***bla***_**TEM-1**_**, *bla***_**AmpC**_	**0.03/4**	**2/4**	** > 512/4**	**0.015/8**	**0.125/8**	**128/8**	**0.06/4**	**0.125/4**	** > 512/4**	**0.007/8**	**0.03/8**	**64/8**
	93,174	*bla*_AmpC_, *bla*_CTX-M-14_, *bla*_OXA-10_	0.25/4	1/4	> 512/4	0.125/8	0.5/8	512/8	0.125/4	0.25/4	> 512/4	0.03/8	0.06/8	128/8
	19–3	*bla*_CTX-M-14_	0.03/4	0.25/4	> 512/4	0.015/8	0.125/8	128/8	0.06/4	0.25/4	> 512/4	0.007/8	0.03/8	64/8
	1564	*bla*_CTX-M-55_, *bla*_CMY-42_, *bla*_TEM-1b_, *bla*_CTX-M-14_	8/4	32/4	> 512/4	4/8	16/8	256/8	0.125/4	0.25/4	> 512/4	0.03/8	0.03/8	128/8
*E. coli*	144,539	*bla*_CTX-M-55_	4/4	4/4	512/4	1/8	2/8	64/8	0.5/4	1/4	512/4	0.25/8	0.25/8	64/8
*K. pneumoniae*	ATCC BAA-1705*	***bla***_**KPC-2**_, ***bla***_**OXA-9**_, ***bla***_**SHV-182**_	**0.125/4**	**1/4**	** > 512/4**	**0.03/8**	**0.125/8**	**64/8**	**0.25/4**	**0.5/4**	** > 512/4**	**0.007/8**	**0.03/8**	**16/8**
	ATCC-700603	*bla*_SHV-18,_ *bla*_OXA-2,_ *bla*_OKP-B-7_	0.03/4	0.25/4	> 512/4	0.015/8	0.06/8	64/8	0.03/4	0.25/4	> 512/4	0.007/8	0.03/8	32/8
	50,666	*bla*_CTX-M-14_, *bla*_KPC-2_	0.25/4	2/4	> 512/4	0.06/8	1/8	256/8	0.5/4	2/4	> 512/4	0.125/8	0.25/8	128/8
	52,582	*bla*_DHA-1_, *bla*_OXA-1_, *bla*_SHV-187_	128/4	256/4	> 512/4	8/8	32/8	512/8	128/4	256/4	> 512/4	2/8	4/8	128/8
	60,700*	***bla***_**DHA-1**_**, *bla***_**SHV-27**_	**0.25/4**	**2/4**	** > 512/4**	**0.125/8**	**0.25/8**	**128/8**	**0.06/4**	**0.125/4**	** > 512/4**	**0.03/8**	**0.03/8**	**64/8**
	61,089*	***bla***_**KPC-2**_**, *bla***_**CTX-M-65**_**, *bla***_**SHV-11**_	**0.125/4**	**2/4**	** > 512/4**	**0.06/8**	**0.5/8**	**64/8**	**0.06/4**	**0.25/4**	** > 512/4**	**0.007/8**	**0.03/8**	**32/8**
	79,528	*bla*_CTX-M-15_, *bla*_SHV-27_, *bla*_TEM-1b_	0.25/4	4/4	> 512/4	0.125/8	2/8	512/8	0.25/4	2/4	> 512/4	0.03/8	1/8	256/8
	47,709	*bla*_SHV-145_	0.125/4	0.125/4	> 512/4	0.06/8	0.06/8	128/8	0.06/4	0.25/4	> 512/4	0.03/8	0.06/8	64/8
	50,604	*bla*_SHV-12,_ *bla*_KPC-2_	0.5/4	0.5/4	> 512/4	0.125/8	0.25/8	64/8	0.06/4	1/4	> 512/4	0.03/8	0.5/8	64/8
*K. pneumoniae*	61,638	*bla*_CTX-M-65,_ *bla*_KPC-2_	1/4	8/4	> 512/4	0.25/8	2/8	32/8	0.125/4	2/4	> 512/4	0.03/8	0.5/8	64/8
	62,291	*bla*_SHV-106,_ *bla*_KPC-2_	0.5/4	2/4	> 512/4	0.125/8	0.5/8	128/8	2/4	2/4	> 512/4	1/8	1/8	64/8
	62,478	*bla*_SHV-110_	0.125/4	0.25/4	> 512/4	0.06/8	0.125/8	64/8	0.06/4	0.25/4	> 512/4	0.03/8	0.06/8	32/8
	78,050	*bla*_SHV106,_ *bla*_KPC-2_	0.5/4	32/4	> 512/4	0.125/8	8/8	128/8	0.125/4	1/4	> 512/4	0.06/8	0.5/8	128/8

CZA, ceftazidime/avibactam; IMR, imipenem/relebactam

Bold font/asterisked isolates were chosen to conduct the time-kill study and the PK/PD study

### Antimicrobial susceptibility test

In accordance with the Clinical and Laboratory Standards Institute (CLSI) recommendations [3], antibiotic susceptibility was determined using the broth microdilution method in Mueller–Hinton broth (Oxoid, Cambridge, UK). And CLSI criteria were used to interpret the results according to the interpretive standards for CZA (: ≤ 8/4 mg/L = sensitive, ≥ 16/4 mg/L = resistant) and IMR (≤ 1/4 mg/L = sensitive, ≥ 4/16 mg/L = resistant). Three different inoculum sizes were used: 10^5^ (standard inoculum), 10^7^, and 10^9^ CFU/mL. Ceftazidime (CAZ, lot: J0100A; potency: 94%), avibactam (AVI, lot: M0321C; potency: 99%) and imipenem (IPM, lot: N1117A; potency: 95%) were purchased from Dalian Meilun Biotechnology Co., Ltd. Relebactam (REL, lot: 002D004; potency: 99.7%) was provided by MSD. *E. coli* ATCC25922 was used as a quality control strain. An inoculum effect was defined as an ≥ eightfold increase in the MIC value upon exposure to a higher inoculum. All tests were carried out in triplicate.

### Time-kill assays

Four strains with the most significant inoculum effect was selected for the time-kill assays. They are *K. pneumoniae* ATCC-BAA 1705 producing KPC-2, OXA-2 and OKP-B-7, *E. coli* 56,706 co-producing TEM-1and AmpC, *K. pneumoniae* 60,700 co-producing SHV-27 and DHA-1 and *K. pneumoniae* 61,089 co-producing KPC-2 and CTX-M-65, respectively. The studies were performed using antibiotics at concentration of 1× , 4× , 16× , and 32× MIC and an initial inoculum size of 10^5^ (standard inoculum), 10^7^ or 10^9^ CFU/mL, respectively. Samples were removed after 0, 1, 2, 4, 6, 8, 12 and 24 h of incubation at 37 °C overnight and plated using an automatic spiral spreading instrument (IUL, Barcelona, Spain) for viable colony counts. All tests were carried out in triplicate.

### In vitro PK/PD studies

#### PK parameters used

The simulated human serum concentrations of CZA and IMR obtained after multiple intravenous administrations were based on PK data from previous studies (Additional file 1: Table S1) [11, 12]. In the present study, a one-compartment PK model of the agents was used for all experiments.

#### In vitro PK/PD simulation model and measurement of antibacterial activity

The study was conducted using the in vitro PK Auto Simulation System 400 (PASS-400; Dainippon Seiki, Kyoto, Japan). The bacterial suspension was injected into 100 mL of broth medium to achieve a initial inoculum of 10^5^, 10^7^ or 10^9^ CFU/mL. At predetermined time points (0, 2, 4, 6, 8, 10, 14, 18, and 24 h), 1.5 mL of the test strain was collected and clones were counted after 18-24 h incubation. Plated using an automatic spiral spreading instrument for colony counts. The limit of clone detection was 30 CFU/mL. Each experiment was performed in triplicate to assure reproducibility.

The PD parameters, including Maximum Kill Down (MKD; the difference between the minimum bacterial count and the initial count during the experiment), the difference in bacterial counts between 0 and 24 h (∆log N24), and the bacterial growth recovery time (RT; the time from first exposure to the antibiotic until the moment when the bacterial count again reached its initial level) were analysed by PASS 400 Analyse Bactericidal Activity software. The area between the control growth curve and bactericidal curves (IE) was calculated by the trapezoidal rule using GraphPad Prism 9; these data were used as the integral parameters for evaluating antimicrobial effects. Data were analysed using one-way analysis of variance, and P < 0.05 was considered statistically significant.

## Results

### Antimicrobial susceptibility tests

When the inoculum size increased from 10^5^ to 10^7^ CFU/mL, the MIC values for CZA against all strains increased by 2- to 64-fold (from 0.03–128/4 mg/L to 0.25–256/4 mg/L), and the MIC values for IMR increased by 1–16-fold (0.03–128/4 mg/L to 0.125–256/4 mg/L). An inoculum effect on CZA and IMR was observed for 52.6% (10/19) and 26.3% (5/19) of isolates, respectively. The most common (by genotype) were SHV and KPC strains. When the inoculum size was 10^9^ CFU/mL, the MIC value for CZA and IMR against all strains was ≥ 512/4 mg/L. When the concentration of AVI and REL was increased from 4 to 8 mg/L, the MIC values for CZA decreased by 2 to  > 16-fold (from 0.03/4 to > 512/4 mg/L to 0.015/8–512/8 mg/L) and the MIC values for IMR decreased by 2 to 64-fold (from 0.03/4 to > 512/4 mg/L to 0.007/8–128/8 mg/L).

### Time-kill assays

**Fig. 1:**
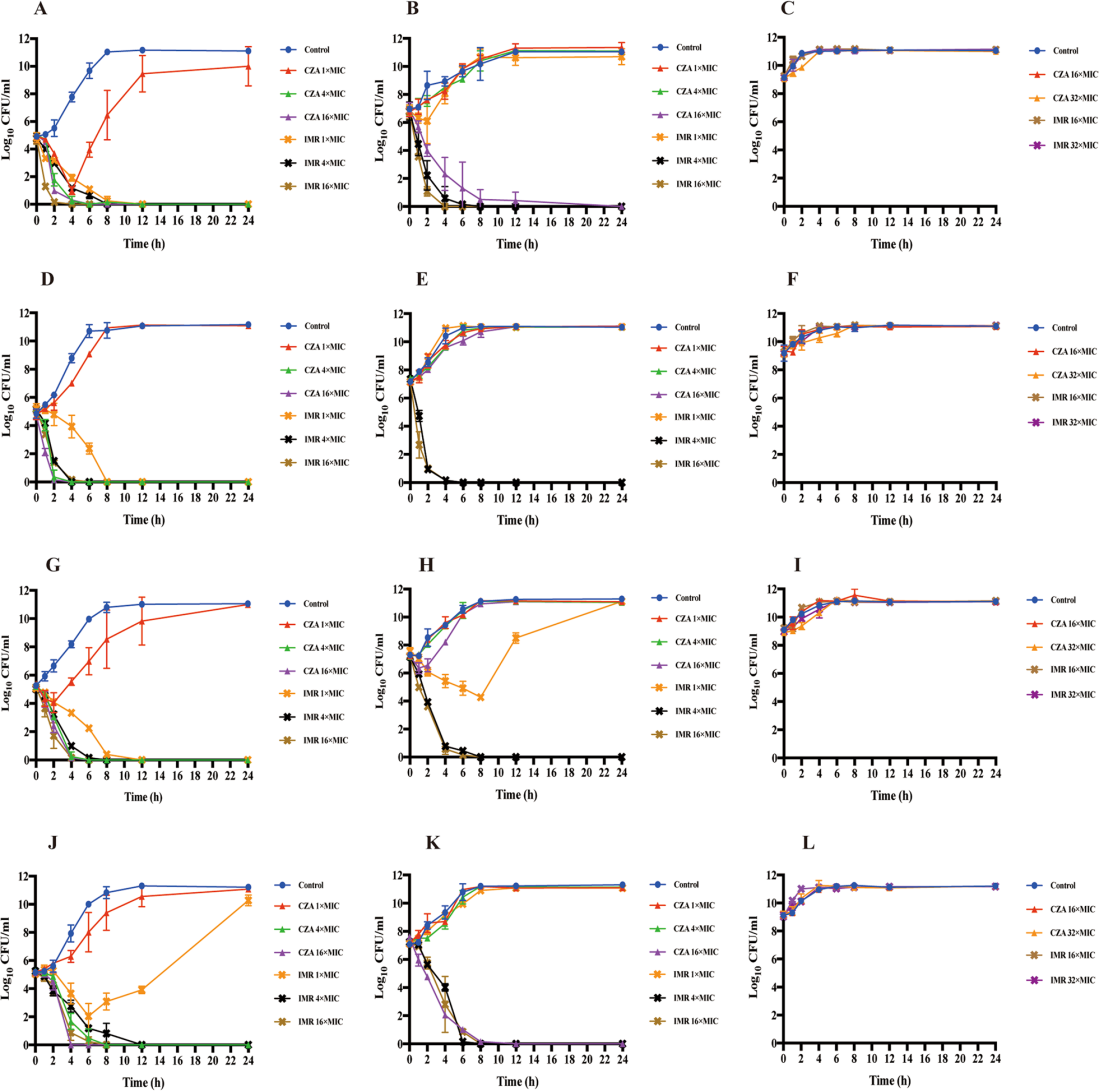
Time-kill curves for different inocula of ATCC-BAA 1705 (**A**–**C**), *E. coli* 56706 (**D**–**F**), *K. pneumoniae* 61089 (**G**–**I**) and *K. pneumoniae* 60700 (**J**–**L**) exposed to CZA and IMR. Data are expressed as the mean ± SD. The left-hand panels depict an inoculum of 10^5^ CFU/mL; the middle panels show an inoculum of 10^7^ CFU/mL; the right-hand panels show an inoculum of 10^9^ CFU/mL. CZA, ceftazidime/avibactam; IMR, imipenem/relebactam

In the presence of the standard inoculum (10^5^ CFU/mL), IMR at 1 × MIC killed 99.9% of *K. pneumoniae* ATCC-BAA 1705(KPC-2-, OXA-9- and SHV-182-producing) and *E. coli* 56,706 (TEM-1- and AmpC-producing) after 8 h, and 99.9% of *K. pneumoniae* 61,089 (CTX-M-65-and KPC-2-producing) after 12 h; these effects were maintained for over 24 h. By contrast, CZA at 1 × MIC led to a < 1log_10_ reduction the CFU/mL of *K. pneumoniae* 60,700 (SHV-27- and DHA-1-producing), but it was unable to match the 99.9% killing effect of IMR. CZA at 4 × MIC killed 99.9% of *K. pneumoniae* ATCC-BAA 1705 and 61,089 at 10^5^ CFU/mL after 6 h, and 99.9% of *K. pneumoniae* 60,700 after 8 h; this effect was maintained for over 24 h. When the inoculum size was increased to 10^7^ CFU/mL, CZA at 16 × MIC killed 99.9% of *K. pneumoniae* ATCC-BAA 1705 and 60,700 after 24 h and 12 h, respectively; however, it had little effect on *E. coli* 56,706 and *K. pneumoniae* 61,089. IMR at 4 × MIC killed 99.9% of the four strains after 6 or 8 h, and maintained this for over 24 h. When the inoculum size rose to 10^9^ CFU/mL, CZA and IMR at high concentrations (32 × MIC) still showed no bactericidal effect against four tested strains (Fig. 1).

### In vitro PK/PD study

**Fig. 2:**
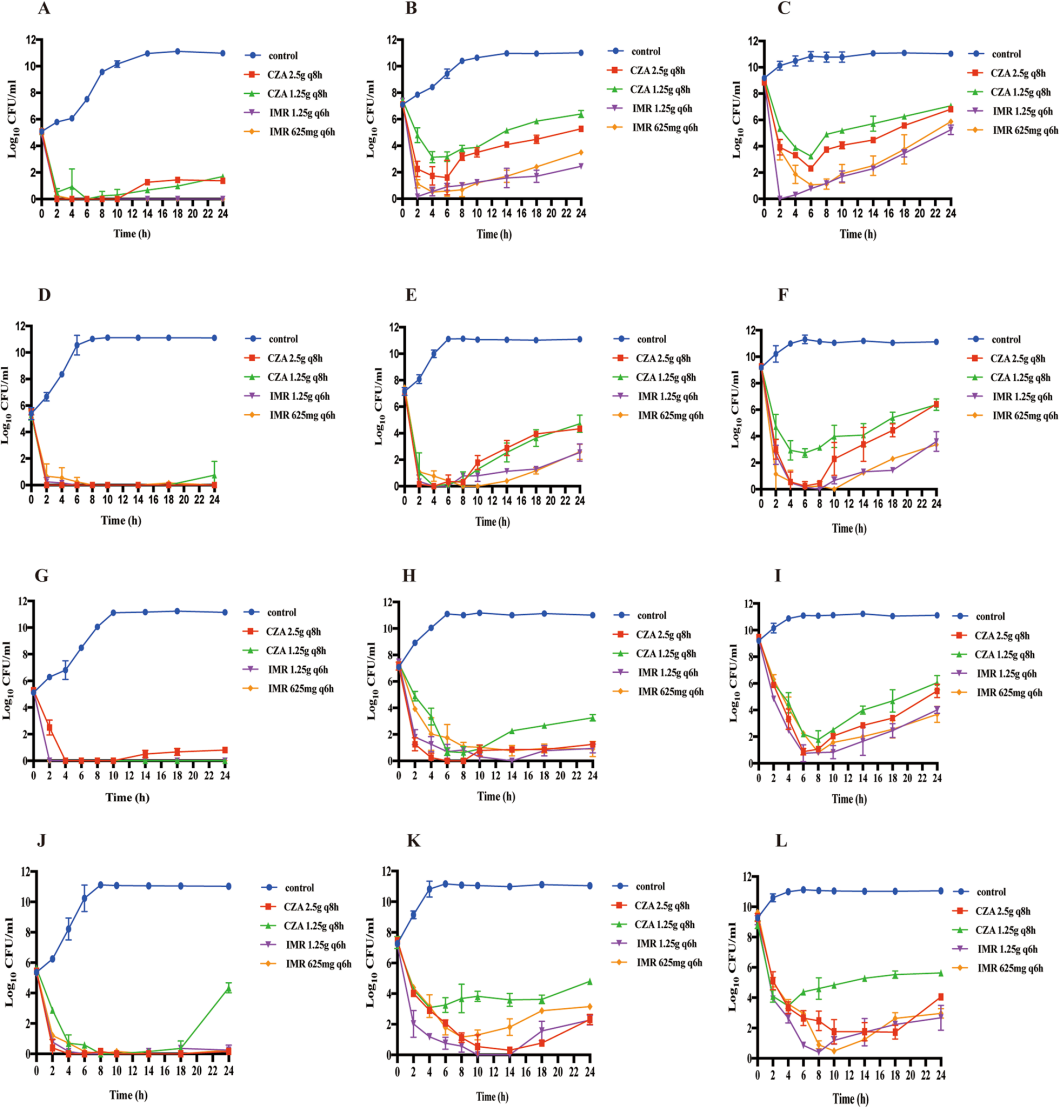
In vitro dynamic time-kill assays for ATCC-BAA 1705 (**A**–**C**), *E. coli* 56706 (**D**–**F**), *K. pneumoniae* 61089 (**G**–**I**) and *K. pneumoniae* 60700 (**J**–**L**). Data are expressed as the mean ± SD. The left-hand panels depict an inoculum size of 10^5^ CFU/mL; the middle panels show an inoculum size of 10^7^ CFU/mL, and the right-hand panels denote an inoculum size of 10^9^ CFU/mL

At an inoculum size of 10^5^ CFU/mL, four dosing regimens (CZA 2.5 g q8h; CZA 1.25 g q8h; IMR 1.25 g q6h; and IMR 625 mg q6h) showed potent bactericidal effects. CZA 2.5 g q8h and IMR 1.25 g q6h killed 99.9% of four strains (*E. coli* 56,706, *K. pneumoniae* ATCC-BAA 1705, 61,089 and 60,700) after 24 h. The killing effects against *E. coli* 56,706 (TEM-1- and AmpC-producing), *K. pneumoniae* 61,089 (CTX-M-65- and KPC-2-producing), and *K. pneumoniae* 60,700 (SHV-27- and DHA-1-producing) were maintained for 24 h; however, bacterial growth resumed after 24 in the presence of CZA 1.25 g q8h. When the inoculum size increased to 10^7^ or 10^9^ CFU/mL, all bacteria recovered after 24 h of exposure to CZA and IMR, although it is noteworthy that regrowth after exposure to CZA was more obvious than that after exposure to IMR (Fig. 2).

**Fig. 3:**
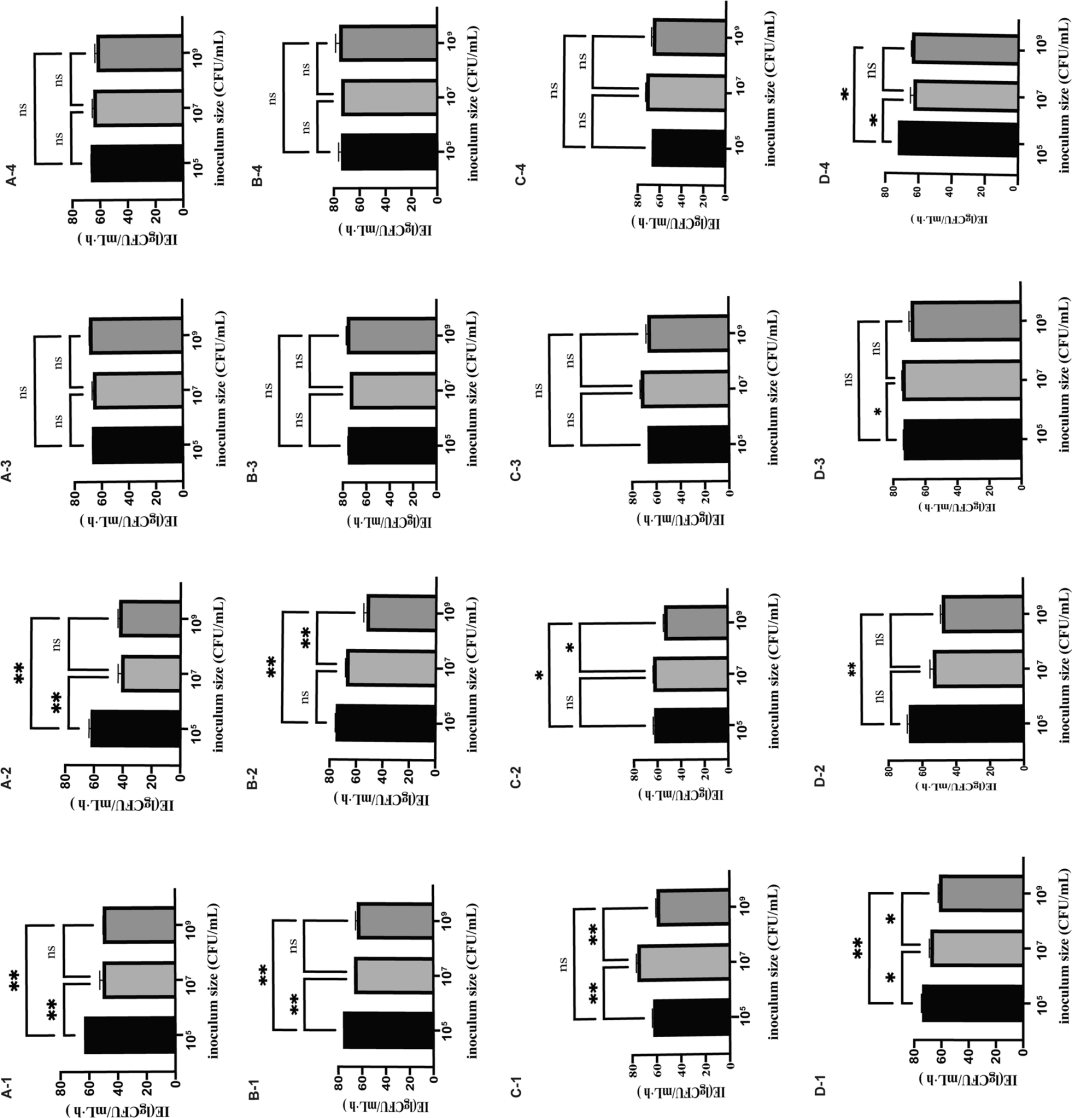
IE of different treatment regimens at different inoculum sizes. ATCC-BAA 1705 (**A**), *E. coli* 56706 (**B**), *K. pneumoniae* 61089 (**C**) and *K. pneumoniae* 60700 (**D**). Data are expressed as the mean ± SD. 1–4 represent different dosing regimens: 1 = CZA 2.5 g q8h; 2 = CZA 1.25 g q8h; 3 = IMR 1.25 g q6h; and 4 = IMR 625 mg q6h

**Fig. 4:**
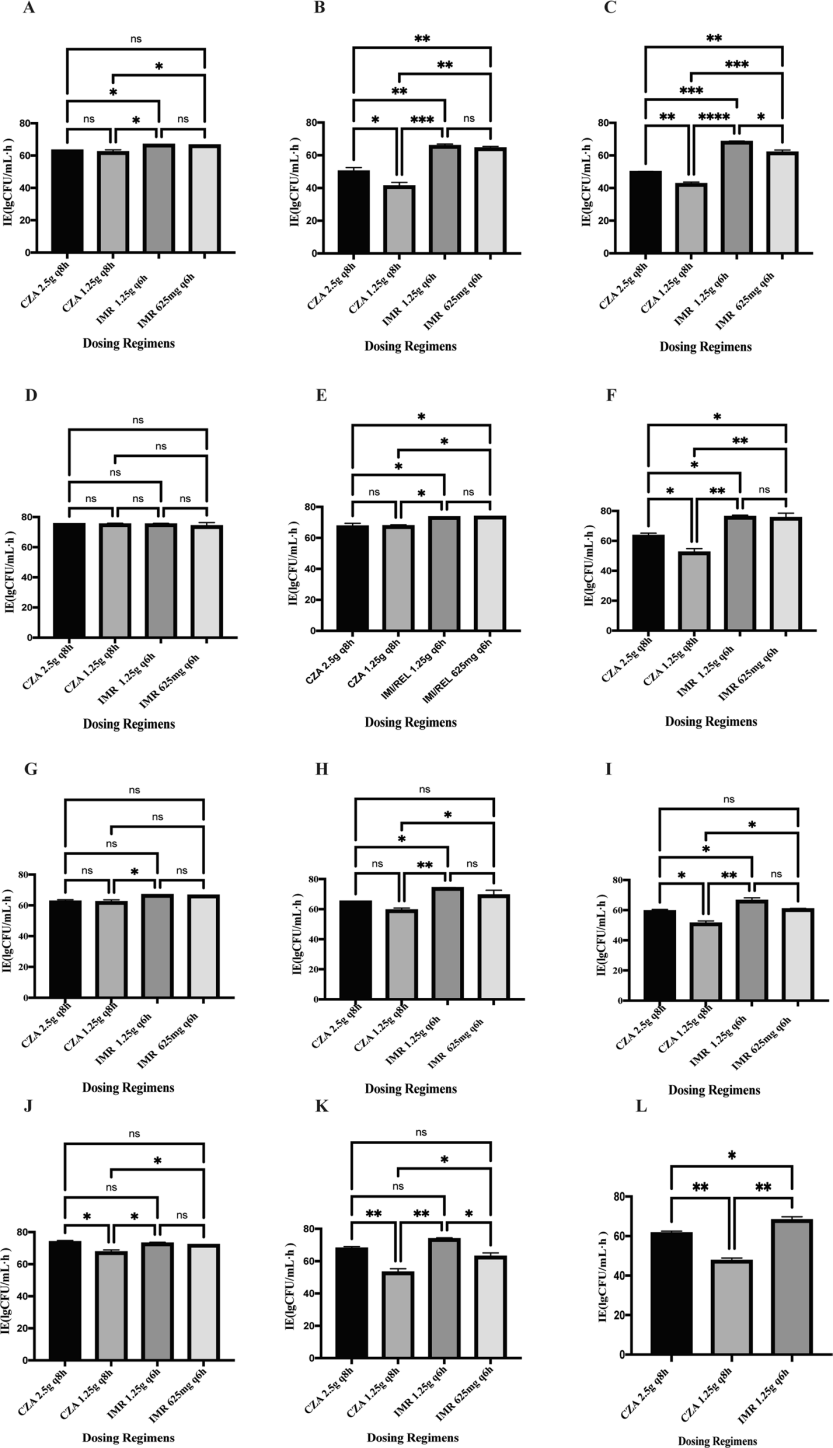
IE of different treatment regimens against four strains at the same inoculum size. ATCC-BAA 1705 (**A**–**C**), *E. coli* 56706 (**D**–**F**), *K. pneumoniae* 61089 (**G**–**I**), and *K. pneumoniae* 60700 (**J**–**L**). The left-hand panels depict an inoculum size of 10^5^ CFU/mL; the middle panels show an inoculum size of 10^7^ CFU/mL, and the right-hand panels denote an inoculum size of 10^9^ CFU/mL

As the inoculum size increased from 10^5^ to 10^7^ and 10^9^ CFU/mL, an obvious bactericidal effect was noted when IMR was administered at 1.25 g q6h. The difference in IE was not significant for any of the inocula (66.79–75.18, 65.77–73.7 and 66.33–76.1 lgCFU/mL∙h, respectively (P > 0.05)). When CZA was administered as 2.5 g q8h, the IE for *K. pneumoniae* ATCC-BAA 1705 was 63.26, 50.24, and 49.99 lgCFU/mL∙h (10^5^ *vs.* 10^7^ CFU/mL [P = 0.007]; 10^7^ *vs.* 10^9^ CFU/mL [P > 0.05]; and 10^5^ *vs.* 10^9^ CFU/mL [P = 0.006]); that for *E.coli* 56,706 was 75.42, 67.47 and 63.47 lgCFU/mL∙h (10^5^ *vs.* 10^7^ CFU/mL [P = 0.008]; 10^7^ *vs.* 10^9^ CFU/mL [P > 0.05]; and 10^5^ *vs*. 10^9^ CFU/mL [P = 0.004]); that for *K. pneumoniae* 61,089 was 62.5, 75.24 and 68.14 lgCFU/mL∙h (10^5^ *vs.* 10^7^ CFU/mL [P = 0.006]; 10^7^ *vs*. 10^9^ CFU/mL [P = 0.03]; 10^5^ *vs*. 10^9^ CFU/mL [P > 0.05]); and that for *K. pneumoniae* 60,700 was 73.83, 67.88 and 61.56 lgCFU/mL∙h, (10^5^ *vs.* 10^7^ CFU/mL [P = 0.03]; 10^7^ *vs.* 10^9^ CFU/mL [P = 0.03]; 10^5^ *vs.* 10^9^ CFU/mL [P = 0.004]). As the inoculum size increased, CZA showed an obvious inoculum effect, although the antibacterial effect of IMR was much more pronounced than that of CZA (Figs. 3, 4, Additional file 1: Table S2).

## Discussion

In recent years, the use of carbapenems to treat severe infections has been increasing worldwide. Under the pressure of antimicrobial selection, the prevalence of carbapenem-resistant bacteria has been increasing year-on-year. Polymyxin, tigecycline and other antibiotics commonly used to treat multidrug-resistant bacterial infections show systemic toxicity and have uncertain efficacy [13]; therefore, new antimicrobial drugs are needed urgently to treat carbapenem-resistant and multidrug-resistant bacterial infections. Classical BLIs such as tazobactam, clavulanic acid and sulbactam show insufficient inhibitory activity against AmpC- or KPC-producing strains. Avibactam, a novel diazabicyclooctanone compound, exhibits potent inhibitory activity against AmpC-, OXA-48- and KPC-producing strains [14], and relebactam also exhibits good activity against SBL-, AmpC- and KPC-producing strains [15]. CZA and IMR show potent antibacterial activity against carbapenem-resistant *Enterobacteriaceae* [16, 17].

The mechanisms underlying the inoculum effect are quite complicated. As the inoculum size increases, the concentration of antibacterial drugs that interact with individual bacterial cells decreases [18], weakening the antibacterial effect of the drugs. Strains with a high inoculum size can reach stationary phase faster, and expression of PBPs during the stationary phase decreases; this weakens the effect of drugs targeting PBPs [19]. At the same time, when the bacterial inoculum size is high, bacterial quorum-sensing can mediate expression of proteins that reduce antimicrobial susceptibility, such as β-lactamases [19]. A previous study found that piperacillin/tazobactam induced a large amount of β-lactamase when the bacterial inoculum size was high [20]. In the present study, as the concentration of BLI AVI and REL increased from 4 mg/L to 8 mg/L, the inoculum effect on CZA decreased from 66.7% (8/12) to 33.3% (4/12), and that on IMR decreased from 25% (3/12) to 8.3% (1/12). As the inoculum size increased, it was necessary to increase the concentration of BLI to retain the antibacterial activity of CAZ and IPM. When the inoculum size was 10^9^ CFU/mL, the MIC values of CZA and IMR were > 512/4 mg/L. In the time-kill assays, even high concentrations of antibiotics (32 × MIC) did not kill the bacteria. Data from the in vitro PK/PD studies showed that the conventional recommended doses of CZA 2.5 g q8h and IMR1.25 g q6h allowed bacterial regrowth after 2–14 h. At a high inoculum size, a large amount of β-lactamase was produced, negating the effects of AVI and REL. CAZ and IPM are hydrolysed by β-lactamases, which reduces their antibacterial effects. Non-β-lactamase-producing *E. coli* ATCC 25922 showed an inoculum effect when the inoculum size increased to 10^9^ CFU/mL, which may suggest that β-lactamase-production is not the only factor involved.

The inoculum effect may also be affected by the type of β-lactamases in β-lactamase-producing strains. In this study, the inoculum effect was greatest against KPC-producing strains and SHV-producing strains. Queenan et al. [21] found that the inoculum effect correlates with the catalytic efficiency of β-lactamase, on the other hand Ehmann et al. [14] stated that the catalytic rate k2/ki of AVI for KPC-2 is 1.3 ± 0.1 × 10^4^ M^−1^ s^−1^. Therefore, the high catalytic efficiency of KPC may be the reason underlying the inoculum effect of KPC-producing strains. A previous study on the inoculum effect of ESBL-producing *E. coli* on piperacillin/tazobactam found no difference in frequency with respect to TEM- producing, SHV- producing, and CTX-M- producing strains [20]. The present study included *K. pneumoniae* but not *E. coli*; therefore, the type of bacteria may have an impact on the presence of an inoculum effect.

An inoculum effect on cephalosporins was observed more frequent than that on carbapenems [18, 19]. A previous study showed that the frequencies of inoculum effect on CAZ, cefepime and cefotaxime were observed for 35%, 85% and 100% of ESBL-producing *E.coli*, respectively, while meropenem did not show an inoculum effect [22]. Another study found that the inoculum effect might attributable to a decrease in expression of penicillin-binding protein (PBP) [23]. CAZ has a higher affinity for PBP3 and IPM mainly binds to PBP2 [24]. When the bacterial inoculum size increases, accumulated signalling molecules such as auto-inducers 2 (AI-2) and Acyl-homoserine lactones (AHLs) mediate quorum-sensing [25]. Then upregulation of β-lactamases expression and downregulation of efflux pump expression and outer membrane protein would led to the reduction of antibiotics susceptibility [25]. The difference in the target protein between CAZ and IPM may be a possible explanation of our finding that an inoculum effect on CZA was observed more frequent than that on IMR.

Many studies have showed that the inoculum effect can impact clinical outcomes [26, 27]. One study found that when the inoculum of *Pseudomonas aeruginosa* increased from 5 × 10^4^ CFU/mL to 5 × 10^5^ and 5 × 10^6^ CFU/mL, the MIC of IMR remained almost unchanged [28]. Here, we found that the frequency of inoculum effect on IMR was relatively low (25%). The clinical efficacy rates of IMR for the treatment of cUTIs and cIAIs are 97.1% and 96.3%, respectively [9, 10], with the difference being non-significant. By contrast, the clinical efficacy rates of CZA for cUTIs and cIAIs are 92% and 80%, respectively [8]. The clinical efficacy of CZA for treating infections at different sites varies greatly, which may be related to the presence of an inoculum effect. We found that the inoculum effect on CZA was 66.7%. However, a previous study suggests that the impact of inoculum size on the in vitro antibacterial activity of CZA is less than that of IMR [29]. This discordance may be due to use of MICs below or above the measurement threshold, making it difficult to analysis MICs statistically. Also, the previous study examined carbapenem-resistant *Enterobacteriaceae*, whereas we tested β-lactamase-producing *E. coli* and *K. pneumoniae*.

Our study has some limitations. First, the experimental strains produced a variety of β-lactamases simultaneously; the actions of these β-lactamases may have affected the antibacterial efficacy of the drugs. Second, we used only conventional recommended regimens (IMR 1.25 g q6h and CZA 2.5 g q8h) and low-dose regimens (IMR 625 mg q6h and CZA 1.25 g q8h) in the in vitro PK/PD study. The efficacy of other regimens (such as high-dose and continuous dosing regimens) on severe infections needs further study.

## Conclusion

IMR and CZA are considered reasonable options for the treatment of multidrug-resistant bacterial infections; however, the presence of an inoculum effect may lead to their failure to treat infections with a high bacterial load (e.g., endocarditis, osteomyelitis, and meningitis); in such cases, IMR may be a better choice. In addition, the presence/absence of an inoculum effect is somewhat determined by the type of β-lactamase. Therefore, the type of β-lactamase should be taken into consideration when selecting antibacterial drugs.

### Supplementary Information

**Additional file 1:** **Table S1.** PK parameters for various dosing regimens. **Table S2**. PD parameters of different regimens against four bacterial strains.

## Data Availability

Genome sequences in this study were submitted to GenBank under the accession Bio Project No. PRJNA1026749.

## Abbreviations

AHL: Acyl-homoserine lactones
AI-2: Auto-inducers 2
AMR: Antimicrobial resistance
AVI: Avibactam
BLBLIs: β-Lactam/β-lactamase inhibitor combinations
CAZ: Ceftazidime
cIAIs: Complicated intra-abdominal infections
CLSI: Clinical and Laboratory Standards Institute
cUTIs: Complicated urinary tract infections
CZA: Ceftazidime/avibactam
ESBL: Extended-spectrum β-Lactamase
IAIs: Intra-abdominal infections
IMR: Imipenem/relebactam
IPM: Imipenem
MKD: Maximum Kill Down
PBP: Penicillin-binding protein
PK/PD: Pharmacokinetics/Pharmacodynamics
REL: Relebactam
RT: Recovery time
UTIs: Urinary tract infections
