# Supplementary material for: Comparison of the inoculum effect of in vitro antibacterial activity of Imipenem/relebactam and Ceftazidime/avibactam against ESBL-, KPC- and AmpC-producing Escherichia coli and Klebsiella pneumoniae
Source: Ann Clin Microbiol Antimicrob. 2023 Dec 10;22:107. doi: 10.1186/s12941-023-00660-5 (PMC10710711; doi:10.1186/s12941-023-00660-5)
Supplement: Supplementary file 1 — Additional file 1: Table S1. PK parameters for various dosing regimens. Table S2. PD parameters of different regimens against four bacterial strains. [file 12941_2023_660_MOESM1_ESM.docx]

**Table S1.** PK parameters for various dosing regimens

| **Dosing Regimens** | **t_1/2_ (h)** | **AUC (mg·h/L)** | **CL (L/h)** | **V_ss_ (L)** | **C_max_ (mg/L)** |
| --- | --- | --- | --- | --- | --- |
| CZA 2.5g q8h | 3.07/2.16 | 198.59/37.41 | 10.07/13.36 | 28.23/25.43 | 93.17/23.33 |
| CZA 1.25g q8h | 1.82/1.79 | 129.28/20.78 | 7.74/12.03 | 18.88/21.08 | 57.35/12.93 |
| IMR 1.25g q6h | 1.1/1.5 | 57.9/36.5 | 15.3/17.3 | 10.4/8.3 | 45.3/21.8 |
| IMR 625mg q6h | 1.0/1.4 | 24.1/15.0 | 13.5/15.3 | 8.6/6.9 | 19.5/9.0 |

**Abbreviation:** CZA, Ceftazidime/avibactam; IMR, Imipenem/relebactam.

t_1/2_, terminal half-life; AUC, area under the plasma concentration-time curve; CL, plasma clearance; V_ss_, apparent volume of distribution at steady state; C_max_, maximum observed plasma concentration.

**Table S2**. PD parameters of different regimens against four bacterial strains

| **Isolates/Dosing regimens** | | **MKD (CFU/mL)** | | | **ΔlgN_24_ (CFU/mL)** | | | | **AUKC (lgCFU/mL∙h)** | | | | | **IE (lgCFU/mL∙h)** | | | |
| --- | --- | --- | --- | --- | --- | --- | --- | --- | --- | --- | --- | --- | --- | --- | --- | --- | --- |
|  |  | **10^5^** | **10^7^** | **10^9^** | **10^5^** | | **10^7^** | **10^9^** | **10^5^** | | | **10^7^** | **10^9^** | **10^5^** | | **10^7^** | **10^9^** |
| ATCC BAA-1705 | CZA 2.5g q8h | -5.49±0.03 | -6.22±0.59 | -7.54±0.42 | -3.67±0.09 | | -1.84±0.30 | -2.05±0.01 | 5.94±0.04 | | | 27.53±2.26 | 35.27±0.16 | 63.26±0.04 | | 50.24±2.26 | 49.99±0.16 |
|  | CZA 1.25g q8h | -5.30±0.04 | -4.47±0.28 | -6.02±0.17 | -3.35±0.11 | | -1.07±0.17 | -2.13±0.43 | 7.0±1.38 | | | 36.69±2.36 | 42.69±1.10 | 62.20±1.38 | | 41.09±2.36 | 42.57±1.10 |
|  | IMR 1.25g q6h | -5.22±0.08 | -7.71±0.12 | -9.55±0.14 | -4.43±0.66 | | -4.94±0.21 | -3.67±0.50 | 2.41±0.06 | | | 12.00±1.08 | 16.82±0.36 | 66.79±0.06 | | 65.77±1.08 | 68.44±0.36 |
|  | IMR 625mg q6h | -5.44±0.45 | -7.02±0.23 | -8.13±0.39 | -4.69±0.05 | | -3.66±0.28 | -3.19±0.37 | 2.83±0.23 | | | 13.47±1.10 | 23.45±1.51 | 66.34±0.23 | | 64.30±1.1 | 61.8±1.51 |
| 56706 | CZA 2.5g q8h | -5.98±0.27 | -7.60±0.31 | -9.25±0.13 | -5.54±0.25 | | -7.10±0.22 | -7.13±0.16 | 2.77±0.13 | | | 15.14±1.97 | 23.66±1.72 | 75.42±0.13 | | 67.47±1.97 | 63.47±1.72 |
|  | CZA 1.25g q8h | -5.73±0.44 | -7.48±0.17 | -6.76±0.31 | -4.58±0.63 | | -5.93±0.09 | -5.87±1.15 | 3.03±0.73 | | 15.03±0.83 | | 34.8±2.50 | 75.17±0.73 | | 67.57±0.83 | 52.32±2.50 |
|  | IMR 1.25g q6h | -5.49±0.03 | -7.55±0.12 | -9.24±0.05 | -5.26±0.21 | | -4.67±0.47 | -5.59±0.74 | 3.02±0.66 | | 9.20±0.26 | | 11.03±1.07 | 75.18±0.66 | | 73.41±0.26 | 76.1±1.07 |
|  | IMR 625mg q6h | -5.50±0.06 | -7.49±0.12 | -9.62±0.31 | -5.29±0.32 | | -4.87±0.70 | -5.89±0.30 | 4.23±2.41 | | 8.87±0.01 | | 11.83±3.20 | 73.96±2.40 | | 73.74±0.01 | 75.29±3.20 |
| 61089 | CZA 2.5g q8h | -5.52±0.10 | -7.27±0.25 | -8.83±0.29 | -5.12±0.08 | | -5.92±0.53 | -4.05±0.70 | 6.77±1.11 | | 8.17±1.43 | | 18.62±0.30 | 62.5±1.11 | 75.24±1.44 | | 68.14±0.29 |
|  | CZA 1.25g q8h | -5.50±0.07 | -7.02±0.15 | -7.79±0.73 | -4.49±0.28 | | -3.91±0.05 | -3.42±0.45 | 7.00±1.38 | | 14.22±0.10 | | 24.95±1.07 | 62.2±1.38 | 68.93±0.45 | | 61.82±1.07 |
|  | IMR 1.25g q6h | -5.64±0.06 | -7.62±0.05 | -8.90±0.21 | -5.23±0.06 | | -6.55±0.16 | -5.24±0.29 | 2.41±0.06 | | 12.7±0.21 | | 20.43±1.84 | 66.79±0.06 | 70.71±0.21 | | 66.33±1.84 |
| 60700 | IMR 625mg q6h | -5.70±0.02 | -6.74±0.29 | -8.57±0.11 | -5.29±0.02 | -6.6±0.47 | | -5.69±0.47 | 2.83±0.23 | 15.67±4.03 | | | 24.13±2.31 | 66.36±0.23 | 67.73±4.03 | | 62.63±2.31 |
|  | CZA 2.5g q8h | -5.71±0.14 | -7.38±0.19 | -7.99±0.65 | -5.29±0.12 | -5.15±0.25 | | -5.24±0.03 | 3.34±0.91 | 16.65±1.17 | | | 25.48±0.97 | 73.83±0.91 | 67.88±1.17 | | 61.56±0.97 |
|  | CZA 1.25g q8h | -5.76±0.24 | -4.43±0.43 | -5.53±0.02 | -1.29±0.49 | -2.53±0.42 | | -3.18±0.25 | 9.64±1.39 | 31.43±2.19 | | | 39.4±1.26 | 67.52±1.29 | 53.1±2.19 | | 47.63±1.26 |
|  | IMR 1.25g q6h | -5.32±0.09 | -7.44±0.03 | -8.79±0.18 | -5.06±0.40 | -4.89±0.52 | | -6.45±1.12 | 4.19±0.74 | 10.83±0.76 | | | 18.84±1.60 | 72.97±0.74 | | 73.7±0.76 | 68.2±1.59 |
|  | IMR 625mg q6h | -5.50±0.17 | -6.09±0.14 | -9.21±0.15 | -5.20±0.45 | -4.04±0.25 | | -6.66±0.37 | 5.04±0.05 | 21.72±2.28 | | | 23.1±0.79 | 72.12±0.05 | | 62.81±2.28 | 63.93±0.79 |

**Abbreviations:** MKD, maximum kill down; AAKC, area above the kill curve; RT, regrowth recovery time; ΔlgN24, bactericidal quantity after 24 h; IE, the area between the control growth and antibacterial killing curves.
